# Quantum Data-Driven Modeling of Interactions and Vibrational Spectral Bands in Cationic Light Noble-Gas Hydrides: [He_2_H]^+^ and [Ne_2_H]^+^

**DOI:** 10.3390/molecules30112440

**Published:** 2025-06-03

**Authors:** María Judit Montes de Oca-Estévez, Álvaro Valdés, Rita Prosmiti

**Affiliations:** 1 Institute of Fundamental Physics (IFF-CSIC), CSIC, Serrano 123, 28006 Madrid, Spain; juditmontesdeoca@iff.csic.es; 2 Departamento de Física, Universidad Nacional de Colombia, Sede Medellín, A. A., Medellín 3840, Colombia; avaldesl@unal.edu.co

**Keywords:** noble gas hydrides cations, advanced quantum-mechanical simulations, electronic structure calculations, machine-learning potential energy surfaces, vibrational spectral transitions

## Abstract

Motivated by two of the most unexpected discoveries in recent years—the detection of ArH^+^ and ${\mathrm{HeH}}^{+}$ noble gas molecules in the cold, low-pressure regions of the Universe—we investigate [He_2_H]^+^ and [Ne_2_H]^+^ as potentially detectable species in the interstellar medium, providing new insights into their energetic and spectral properties. These findings are crucial for advancing our understanding of noble gas chemistry in astrophysical environments. To achieve this, we employed a data-driven approach to construct a high-accuracy machine-learning potential energy surface using the reproducing kernel Hilbert space method. Training and testing datasets are generated via high-level CCSD(T)/CBS[56] quantum chemistry computations, followed by a rigorous validation protocol to ensure the reliability of the potential. The ML-PES is then used to compute vibrational states within the MCTDH framework, and assign spectral transitions for the most common isotopologues of these species in the interstellar medium. Our results are compared with previously recorded values, revealing that both cations exhibit a prominent proton-shuttle motion within the infrared spectral range, making them strong candidates for telescopic observation. This study provides a solid computational foundation, based on rigorous, fully quantum treatments, aiming to assist in the identification of these yet unobserved He/Ne hydride cations in astrophysical environments.

## 1. Introduction

Molecular spectroscopy is a fundamental tool for investigating molecular structures, properties, dynamics, and chemical reactivity. However, spectral data do not directly reveal molecular information; instead, it is encoded in complex patterns that require careful analysis. To extract meaningful insights, computational modeling plays an essential role in decoding these spectra. By combining advanced methods and techniques, such as quantum chemistry calculations, molecular dynamics simulations, and machine learning algorithms, computational spectroscopy allows researchers to simulate and interpret spectral features, enabling a deeper understanding of molecular behavior. Recent advancements in computational methods have significantly expanded the applicability of such approaches, facilitating, in this way, direct comparisons with experimental data [1,2,3,4,5,6,7,8]. A computational model is built upon specific concepts and assumptions, ideally adaptable to any molecular system. Quantum mechanical approaches, in particular, rely on the molecular Hamiltonian operator, incorporating both kinetic and potential energy, to extract vibrational information. Achieving high accuracy in spectral simulations requires both rigorous nuclear quantum simulations and high-level electronic structure calculations to provide an accurate description of interaction energies and an efficient representation of the potential energy surface (PES). The PES is a cornerstone for understanding most molecular processes, and its precise representation is crucial for reliable spectral computations. Commonly, discrepancies between theoretical predictions and experimental observations arise from inaccuracies in the underlying potential representation. As molecular complexity increases, refining PES models becomes progressively more challenging.

This study focuses on computing molecular interactions and characterizing the vibrational transitions of cationic light noble-gas hydrides of astrophysical interest. For a long time, the interstellar medium (ISM) has been considered an inhospitable environment to chemical compounds due to the extreme conditions that characterize it, and only a few molecules can be formed, but recently over 300 molecules have now been identified [9]. Notably, the discovery of noble gas hydride cations like HeH^+^ in the planetary nebula NGC 7027 [10] and ArH^+^ for first time in Crab Nebula [11], and later, in extragalatic sources [12], challenges the notion of noble gases as inert, indicating the need of further research for a much better understanding of the noble gas chemistry.

Among the cationic noble-gas hydrides, the most astrophysically relevant are those of helium and neon. The interest in [He_2_H]^+^ lies in its composition of helium and hydrogen, the two most abundant elements in the universe, increasing the likelihood of its formation in suitable space environments. The fifth most abundant element is neon, and thus it is also an intriguing candidate. However, no molecular species containing it has been detected so far, the NASA’s LADEE mission recently confirmed [13] the presence of neon in the lunar exosphere, suggesting that neon hydrides or their cations could exist in cold planetary systems under favorable conditions for Ne interactions with H or H^+^.

In the laboratory, high-resolution mass spectrometry experiments [14,15,16,17] have provided insights into the stability of specific structures, revealing differences with those observed in heavier noble gas hydrides, [Ng_*n*_H_*m*_]^+^ (*m* = 1, 2 and 3). Thus, noble gas matrix experiments have confirmed the existence of centrosymmetric [NgHNg]^+^ configurations [18,19,20,21], highlighting that the symmetric insertion of a proton into a weakly bound noble gas dimer significantly enhances the system’s stability compared to other possible arrangements. Just recently, applying different action spectroscopic techniques [8,22] the $\nu_{3}$ proton shuttle motion of He_2_H^+^ has been observed at 1315.84 cm^−1^, together with seven of its rovibrational lines [8], while estimates for the bending and symmetric stretch fundamentals have also been reported [8,22,23] at 874.9 and 956.6 cm^−1^, respectively.

Several theoretical studies have been also carried out predicting both geometric and energetic characteristics of the linear configurations of these systems, identifying the centrosymmetric structure as the most stable [24,25,26,27,28,29,30,31,32,33,34,35,36,37,38,39]. In particular, for the [He_2_H]^+^ case, Dykstra [27] has first computed potential energy values for a limited set of geometries, which later on, Lee and Secrest [37] have used to parameterize an analytical PES and calculate rotational-vibrational states up to *J* = 2. They have identified five bound states for *J* = 0 for both [He_2_H]^+^ and [He_2_D]^+^. Subsequently, Baccarelli et al. [28] have explored the potential for both collinear and C_2_ symmetry geometries using MRCI/pVTZ calculations, while another significant study was carried out by Kim and Lee [29], examining a restricted region of the configuration space with the CCSD(T) method. In 2003, Panda and Sathyamurthy [34] constructed a PES using the many-body expansion form [40] fitted to data from CCSD(T)/AVTZ calculations, and reported seven and fourteen bound vibrational states for the [He_2_H]^+^ and [He_2_D]^+^, respectively. Later, Liang et al. [38] developed another analytical PES using 15682 MRDCI/AV5Z data points up to energies of 10000 cm^−1^. More recently, Stephan and Fortenberry [32] reported quartic force fields derived from CCSD(T)/AV5Z calculations, while Fortenberry and Wiesenfeld [33] reported an updated PES using CCSD(T)-F12 data. In contrast, for the [Ne_2_H]^+^ complex, the literature is more limited. There are only two PESs available: a quartic force-field PES [31] using CCSD(T)/AV5Z calculations, and an analytical 3D PES [35] constructed from more than 23000 CCSD(T)/AVQZ data points. In this latter study, the first 22 vibrational bound levels have also been reported for [Ne_2_H]^+^ [35].

Advances in computational quantum chemistry coupled with cutting-edge experimental techniques have opened new avenues to explore the fundamental chemical properties of these species. A common aspect in all previously mentioned computational studies is the use of traditional analytical PES models. While such models have demonstrated the ability to provide high-quality results [41,42,43,44,45,46,47,48], even today, despite the feasibility of high-level quantum chemistry calculations, their construction still remains a laborious and time-consuming computational task. In this context, machine learning (ML) PESs trained on previously calculated molecular structures and energies [49,50,51], present a promising alternative [52,53,54,55,56,57] for studying various physical-chemical processes, e.g., the vibrational analysis of molecules. In our recent investigations [58,59,60,61], we have assessed the performance of machine learning PESs methods based on both kernel and neural networks (NN) representations [55,62,63,64,65,66]. Specifically, for small size systems, the reproducing kernel Hilbert space (RKHS) method [67,68,69] has proven effective, by constructing accurately and reproducing the training data, while simultaneously capturing potential’s long-range asymptotic interactions using appropriate kernel polynomials [70,71,72,73,74,75].

Therefore, in this work, we aim to develop a full three-dimensional kernel-based RKHS ML-PES for the [He_2_H]^+^ and [Ne_2_H]^+^ noble gas hydride cations. These PES models will be trained and validated against high-accuracy CCSD(T)/CBS data, with each step of the PES construction process being thoroughly examined. The resulting ML-PES will be utilized in multi-configuration time-dependent Hartree (MCTDH) ro-vibrational quantum computations to provide precise predictions of energetic and spectroscopic properties for common isotopologues, which will then be compared with available literature data. The calculated vibrational transitions could provide valuable insights for supporting the astrochemical identification of these noble gas compounds in unexplored regions of the interstellar medium.

## 2. Results, Discussion and Computational Methods

### 2.1. Electronic Structure Calculations: Reference Data on Interaction Energies

All ab initio electronic structure calculations were performed using the MOLPRO 2022 program [76], while the DENEB software package [77] was employed to generate and organize all input and output data files, respectively.

#### 2.1.1. Optimized Structures and Dissociative Energetics

**Figure 1:**
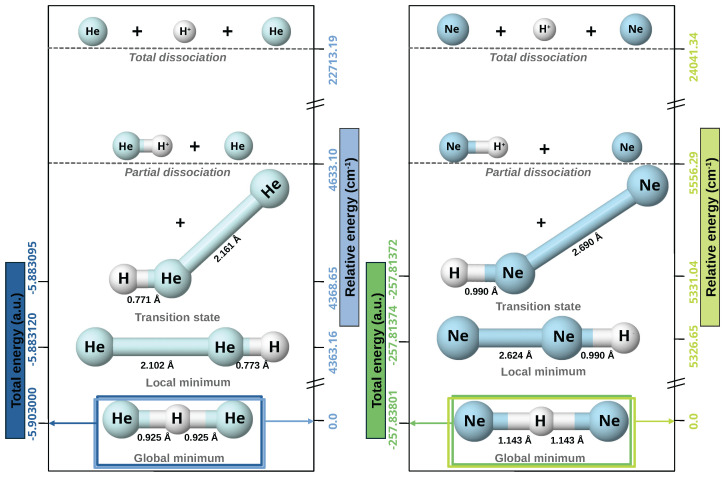
Optimized energy structures (global and local minima, transition state) together with their partial and total dissociation energies for the [He_2_H]^+^ (**left panel**) and [Ne_2_H]^+^ (**right panel**) from the CCSD(T)/AV6Z computations.

We have first carried out geometry optimizations of these complexes at the CCSD(T)/aug-cc-pV6Z level of theory, together with the corresponding harmonic vibrational normal modes frequency analysis. Figure 1 summarizes both energies and configurations of stationary points and dissociation channels. We have found that the optimized equilibrium structure (global minimum) of both triatomic complexes shows a Ng–H^+^–Ng (linear) configuration, belonging to $D_{\infty h}$ symmetry, with the Ng–H^+^ bond lengths of 0.925/1.143 Å and a total energy of −5.9030/−257.8380 a.u. for He/Ne systems. We can note that the Ng–H^+^ bond lengths are larger compared with 0.77/0.90 Å in the isolated [NgH]^+^ molecules [78] due to the incorporation of the new Ng atom. We also found that the Ng–Ng–H^+^ (antilinear) optimized structure (local minimum) is at an energy of −5.883120/−257.8137 a.u., and 4367.16/5326.65 cm^−1^ above the global one, while the barrier between them is found at an energy of −5.883095/−257.81372 a.u, or 4368.64/5331.039 cm^−1^.

The harmonic vibrational frequencies for the ^4^He–H^+^-^4^He and ^20^Ne–H^+^-^20^Ne from the CCSD(T)/AV6Z computations are shown in Table S1 (see Supplementary Material), where $v_{1}$ is the symmetric Ng–H^+^ stretch, $v_{2}$ the doubly degenerate proton bend vibration, and $v_{3}$ the antisymmetric Ng–H^+^ stretching or shared-proton mode. The calculated harmonic zero-point-energy (ZPE) values are 2295.065 cm^−1^ for [He_2_H]^+^ and 1782.385 cm^−1^ for the [Ne_2_H]^+^, with the harmonic vibrational frequencies being in line with the reported values in previous studies [31,32,36]. Regarding the corresponding intensities, those of shared-proton stretching modes have higher values compared to $v_{2}$ modes, indicating a strong absorption or emission line in the IR spectra. Furthermore, it can be seen that the proton-shuttle motion is very similar in both species, with a difference of just 3 cm^−1^. We also note that, in the case of [He_2_H]^+^, the trend in frequencies observed in its heavier counterparts is not followed, where $v_{1}$ < $v_{2}$ < $v_{3}$ is the rule, instead, the order is partially inverted ($v_{2}$ < $v_{1}$ < $v_{3}$) and the value of $v_{1}$ is twice that in the Ne-complex, and almost six times larger than in the [Ar_2_H]^+^ [59].

Next, we also performed CCSD(T)/AV6Z computations to determine the energetics associated with the possible dissociation channels, as shown in Table S2 (see Supplementary Material). Based on our calculations, the most favorable direct dissociation pathway for [Ng_2_H]^+^ complexes involves the separation into a neutral Ng atom and NgH^+^ molecular cation. The relative energy associated with this process is 4633.10 cm^−1^ for He-complex and 5556.29 cm^−1^ for the Ne-complex (see Figure 1). The next lowest dissociation energy corresponds to the loss of the proton, which requires an energy five times greater than the predecessor dissociation channel. In this case, a competition is observed between the formation of Ng_2_ and the dissociation in 2Ng, with an energy difference of less than 1650 cm^−1^ in both scenarios. For [He_2_H]^+^, the most favorable channel is the formation of Ng_2_, while in the case of [Ne_2_H]^+^ the opposite mechanism is favored. A similar behavior is presented in the dissociation mechanism by the loss of a hydrogen atom, with an energy twenty-three and fifteen times greater than the most favorable dissociation mechanism for He-complex and Ne-complex, respectively. However, in this case, the energy difference between both pathways is much smaller, being approximately 15 cm^−1^ for [He_2_H]^+^ and 12 cm^−1^ for [Ne_2_H]^+^. These significant energy differences strongly indicate that the predominant formation method for these complexes is through the generation of the NgH^+^ and Ng atoms. Consequently, our study of their interaction will focus on their dissociation into this lowest-energy channel.

In this context, Table S3 (see Supplementary Material) presents the stepwise formation energies as Ng atoms bind to the proton, revealing that the first Ng atom binds more strongly than the second in all studied systems. The total formation energy at T = 0 K for the [He_2_H]^+^ and [Ne_2_H]^+^ systems is obtained by summing the stepwise formation energies, with the first binding step playing a dominant role in determining the overall behavior. For He_2_H^+^, in general terms, an excellent agreement with the data available in the literature is observed. Compared with data in ref. [33], the discrepancies of almost 6 kcal/mol in the total formation energy and 1.5 kcal/mol in the second step are mainly due to the inclusion of zero-point energy corrections, which were not considered here, while the difference of approximately 1 kcal/mol with ref. [28] is due to the use of a different computational method (MRDCI). In the case of Ne_2_H^+^, the observed discrepancies are again due to the differences in the computational methods used [35,39,79]. Comparing with the most recent study [35] a difference of about 1 kcal/mol is observed, suggesting that for noble gas hydrides, the accuracy of the calculations becomes increasingly dependent on the size of the basis used as the Ng atom becomes heavier, as already observed in ref. [58,78].

#### 2.1.2. Training and Testing Datasets

We have used the Jacobi’s coordinates to sample the configuration space for both [He_2_H]^+^ and [Ne_2_H]^+^ cations. The Jacobi (*r*, *R*, θ) coordinates are defined as *r* the vector along the Ng–H bond distance, *R* the vector along the distance between Ng from the center of mass of the H, and θ the angle between the (*r*, *R*) vectors. On the basis of our previous works [59,78], we have employed the single and double excitation coupled cluster with the perturbative triples (CCSD(T)) method using the AV5Z and AV6Z basis sets, followed by extrapolation to the complete basis sets (CBS) to obtain the energies. We have utilized the two-point single inverse power function introduced by Schwartz [80] $E_{n}^{corr}$ = $E_{CBS}^{corr}$ + $\frac{A}{n^{3}}$, with *n* = 5 and 6, in order to compute the correlation energy at the CBS limit, and then used it in the E_CBS_ = E_HF/AV6Z_${\mathrm{+E}}_{CBS}^{corr}$ calculation.

**Figure 2:**
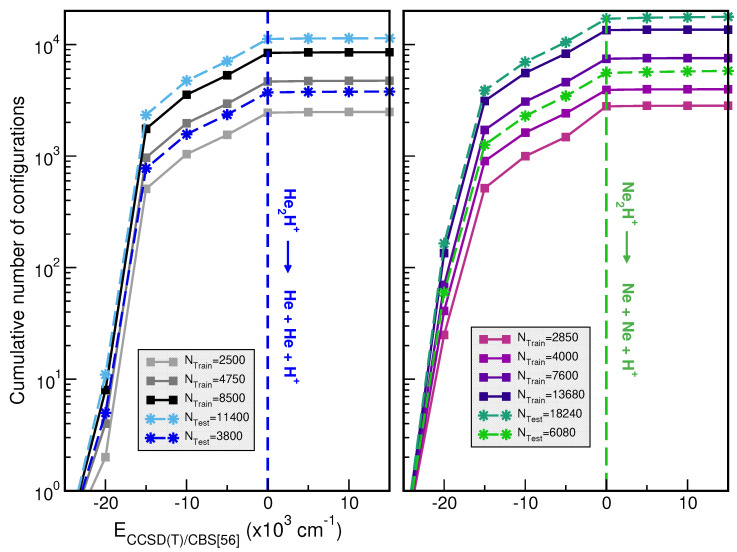
Cumulative number of configurations vs CCSD(T)/CBS[56] energies for the indicated training and testing set sizes for [He_2_H]^+^ (**left panel**) and [Ne_2_H]^+^ (**right panel**). The dashed lines indicate the NgH^+^ + Ng dissociation thresholds.

The size of the training datasets was up to 8550 and 13680 CCSD(T)/CBS[56] ab initio points for the [He_2_H]^+^ and [Ne_2_H]^+^ molecular cations, respectively. The [He_2_H]^+^ dataset was distributed in a grid of 10-points in the *r* coordinate ranging between 0.60 and 1.95 Å, 45-points in the *R* distance from 1.5 to 7.0 Å, and 19-points in the angular θ coordinate between 0 and 180^∘^, while for [Ne_2_H]^+^ the grid is ranging from 0.75 to 2.25 Å, and 1.9 to 7.0 Å in *r* and *R* coordinates, respectively. For both molecules, we employed eight different sets of training data with, in total, 1000(1900)/1500(2850), 1800(3420)/2700(5130), 2500(4750)/4000(7600) and 4500(8550)/7200(13680) points by choosing 4 or 10/6 or 16, 25 or 45, and 10 or 19 grid points in the *r*, *R* and θ descriptors, respectively, for the [He_2_H]^+^/[Ne_2_H]^+^, keeping the same coordinate range in all of them. Once the generation of a high-quality dataset is completed, we then proceed with its organization by splitting the whole dataset into training and test sets, which are then utilized in the training and evaluation processes, respectively. In Figure 2, we show the distributions of the cumulative number of configurations as a function of energy for the previously mentioned training sets, as well as, 11400 and 1840 additional testing dataset configurations of He- and Ne-complexes. We have also shown the distribution of energy of 3800 and 6080 randomly chosen data for each [He_2_H]^+^ and [Ne_2_H]^+^, respectively.

### 2.2. Potential Energy Surface Representations: Topology and Quality

#### 2.2.1. RKHS ML-PESs Methodology

As mentioned, we choose the RKHS method proposed by Ho and Rabitz [67] to represent the PESs (see also ref. [52] and references therein). Briefly, V(x) represents the potential energy function, with $V_{i}$ denoting the known potential energies at specific molecular configurations $x_{i}$, then, by applying the representer theorem to a general functional relationship, V(x) can be optimally approximated as a linear combination of appropriate functions: $\tilde{V}\left(x\right)\approx {\tilde{V}}_{i}=\sum_{i=1}^{N}C_{i}K\left(x,x_{i}\right)$, with $C_{i}$ coefficients and $K(x,x^{\prime })$ a kernel function. The coefficients $C_{i}$ are determined by solving the linear equation $C=K^{-1}y$, where $y=[V_{1},V_{2},\ldots ,V_{N}]$ represents the input data, $C=[C_{1},\ldots ,C_{N}]$ is the vector of coefficients, and K is the N×N reproducing kernel matrix. Furthermore, the multidimensional kernel function $K(x,x^{\prime })$ can be expressed as a direct product: $K\left(x,x^{\prime }\right)=\prod_{d=1}^{D}k\left(x,x^{\prime }\right)$, where *D* is the dimensionality of the kernel *K*, and *k* represents the one-dimensional (1D) kernels. Several 1D kernel functions for different purposes are available in the literature, and in this study, we have employed the $k_{1}^{n,m}$, $k_{2}^{n,m}$ and $k_{3}$ 1D reproducing kernel functions for the diatom distance-like (*r*), the Ng-diatom distance-like (*R*), and angle-like (θ) variables, respectively. In the above equation, the reduced coordinate is z=cosθ, whereas, $N_{r}$, $N_{R}$ and $N_{\theta }$ are the number of ab initio calculated points in each coordinate. The reproducing distance-like kernel functions $k_{1}$ and $k_{2}$ are represented by $k_{1,2}^{n,m}=n^{2}\chi_{>}^{-(m+1)}{B(m+1,n)}_{2}F_{1}\left(-n+1,m+1;n+m+1;\frac{\chi_{<}}{\chi_{>}}\right)$, with χ= *r* or *R*, respectively. $\chi_{>}$ and $\chi_{<}$ are the larger and smaller of the χ, respectively, while the angle-like kernel function is given as $k_{3}\left(y,y^{\prime }\right)=\sum_{l}\frac{\left(2ll+1\right)}{2}P_{l}\left(y\right)P_{l}\left(y^{\prime }\right)$. The *n* and *m* superscripts refer to the order of smoothness of the function and its asymptotic behavior at large distances, with n=2, and *m* = 3 accounting for the $R^{-4}$ leading dispersion interaction between the Ng atom and NgH^+^ molecular ion. *B* is the beta function, F12 is the Gauss hyper-geometric function, and P_*ll*_ the Legendre polynomials with ll = 0–18. In turn, the potential form is given by

(1) $$V\left(r,R,\theta \right)=\sum_{i=1}^{N_{r}}\sum_{j=1}^{N_{R}}\sum_{k=1}^{N_{\theta }}C_{ijk}\;k_{1}^{n,m}\left(r_{i},r\right)\;k_{2}\left(R_{j},R\right)\;k_{3}\left(\theta_{k},\theta \right).$$

#### 2.2.2. Validation of the RKHS ML-PES Models

A three-step validation protocol previously developed [59] was employed as follows: First, each PES’s model is validated on the training data to ensure that the results are consistent and reproducible with the information used during training. Then, potential values are generated at points outside the training range, using the test sets, to assess the ability of the model to generalize to new and unseen data. Finally, regression diagnostics are monitored. Here, we will focus on the root-mean-square error (RMSE) and mean-absolute error (MAE), and it is verified that they do not exceed a predefined threshold. If the error exceeds this limit, more training data are incorporated to improve the model’s accuracy.

**Figure 3:**
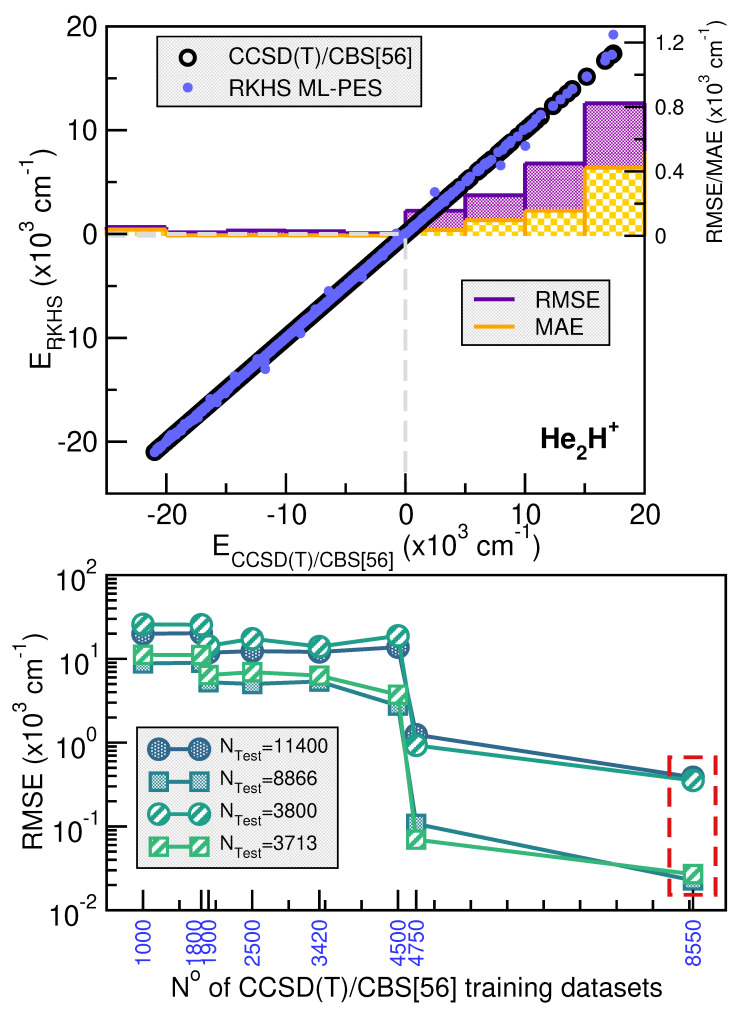
Correlation plots comparing the performance of the RKHS ML-PES with respect the reference CCSD(T)/CBS[56] testing set energies (**upper panel**) of [He_2_H]^+^ cation. The averaged RMSE and MAE values are also shown as a function of CCSD(T)/CBS[56] energy ranges (**lower panel**). The total average RMSE values of the RKHS models vs. the number of training and testing datasets are plotted in the **lower panel**.

**Figure 4:**
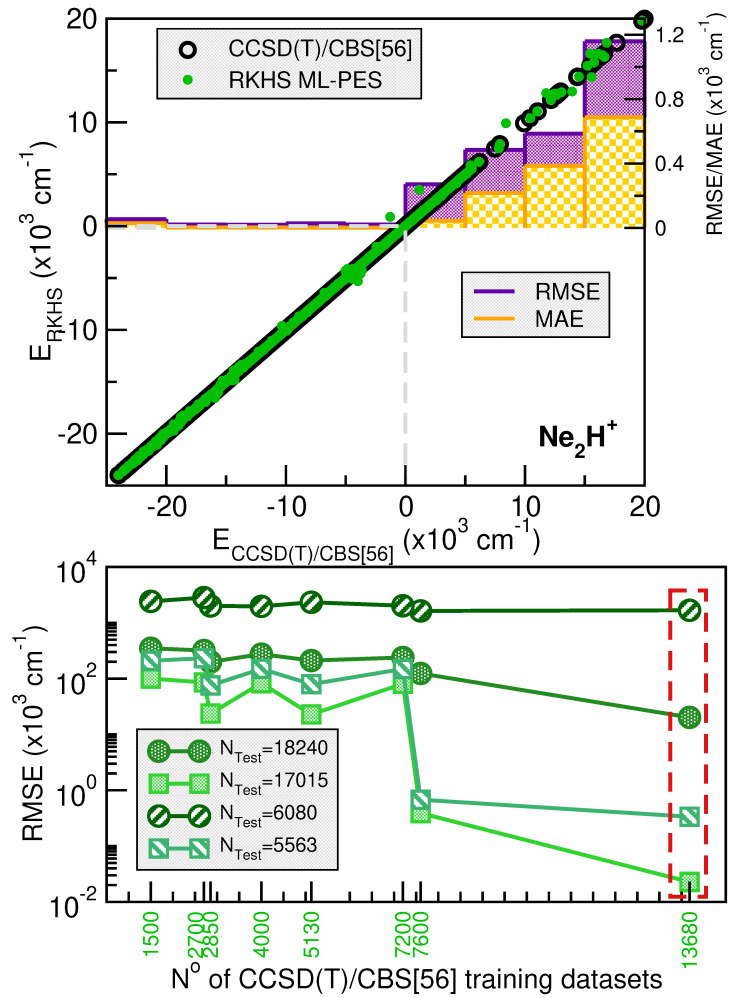
Same as Figure 3 for the [Ne_2_H]^+^ cation.

Figure 3 and Figure 4 illustrate the systematic improvement in the RMSE values of the RKHS ML-PES model as the number of training data increases. To select the best-performing RKHS PES for each complex, we employed a hold-out cross-validation scheme among the eight trained models. The RMSE values were computed considering the total number of testing data for [Ng_2_H]^+^ cations. In the case of the He-complex, we averaged the RMSE over 3800 randomly selected points from the total 11400 test data, while for the Ne-complex, we used 6080 random points out of 18240. This allows us to assess the quality of the RKHS PES as a function of the training data size, with models trained on 4500/8550, 2500/4750, 1800/3420, and 1000/1900 or 7200/13680, 4000/7600, 2700/5130 and 1500/2850 points for both He or Ne-complexes (shown in the left and right plots of Figure S1 in Supplementary Material, respectively). As expected, RMSE values decrease as the dataset size increases. Additionally, we observed that RMSE values are highly sensitive to the sampling in the θ coordinate. Specifically, models trained on 4500/7200 (10, 45, 10/16, 45, 10), 2500/4000 (10, 25, 10/16, 25, 10), 1800/2700 (4, 4, 10/6, 45, 10), and 1000/1500 (4, 25, 10/6, 25, 10) exhibit significantly higher RMSE values compared to models trained on 8500/13680 (10, 45, 19/16, 45, 19), 4750/7600 (10, 25, 19/16, 25, 19), 3420/5130 (4, 45, 19/6, 45, 19), and 1900/2850 (4, 25, 19/6, 25, 19), where the RMSE values are significantly lower. The computed RMSE values for all RKHS ML-PES models, considering the total 11400/18240 test data, the 8866/17015 datasets with energies below the dissociation threshold, and the two corresponding randomly selected testing sets 3800(3713)/6080(5563), follow the same trend.

The correlation plots demonstrated the effectiveness of the selected RKHS ML-PES model by comparing its performance against 8500/13680 training and 11400/18240 testing data points for He/Ne molecular cations, covering both the attractive and repulsive regions of the potential. The RMSE values, shown as a function of energy ranges (see upper panels of Figure 3 and Figure 4), indicate that outside the training region, they remain at 22/23 cm^−1^, even for dissociation energies exceeding 8866/17015 configurations (see lower panels of Figure 3 and Figure 4). Additionally, the total mean absolute error (MAE) remains below 0.004% across 11400/18240 configurations.

When we analyze the overall quality of the RKHS ML-PES developed in this work, it can be seen that the amount of training data required for their construction is significantly lower compared to previous studies available in the literature for both complexes analyzed, such as those reported in [35,38]. For the case of [He_2_H]^+^, there are two PESs in which RMSE values with respect to the ab initio data of 87.44 and 17.1 cm^−1^ are reported, references [34,38], respectively. The last one of these models used 15682 points with energies below 10000 cm^−1^, which contrasts with the non-error of the RKHS ML-PES presented here. In the case of [Ne_2_H]^+^, the study by Koner et al. [35] reports an RMSE of 9.09 cm^−1^, based on more than 23000 potential energy values computed ab initio. In contrast with all of this, the RKHS ML-PESs developed in this work require a considerably smaller training set, demonstrating the efficiency of the kernel-based approach in the study cases. This approach significantly reduces the amount of data required to build a high-precision PES compared to traditional analytical methods, as evidenced by recent studies.

Apart from the typical quantitative error analysis of the RKHS ML-PES, we also proceed to analyze the behavior of the hydride cations under study through their potential curves plots along representative coordinates.

**Figure 5:**
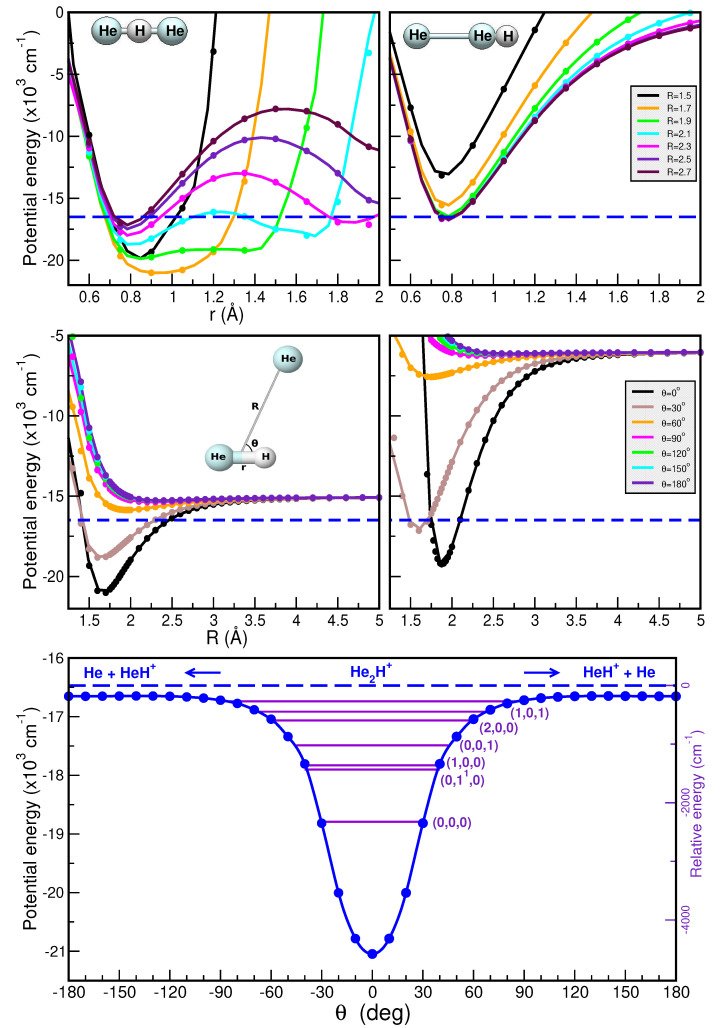
Potential curves for the [HeHHe]^+^ complex as a function or *r* (**upper panels**) in its linear He–H^+^–He (**left panel**) and antilinear He–He–H^+^ (**right panel**) configurations with the *R* distance fixed at the indicated values, and as a function of *R* (**middle panels**) for the indicated θ values for *r* = 0.9 Å (**left panel**) and 1.35 Å (**right panel**). Minimum energy path values as a function of θ angles (**lower panel**) are plotted for the [He_2_H]^+^ with the corresponding vibrational energy levels ($\nu_{1}$, $\nu_{2}$, $\nu_{3}$) from the MCTDH calculations superimposed. The CCSD(T)/CBS[56] energies are shown in solid circles, while the RKHS ML-PES values are shown in solid lines. The dashed blue lines indicate HeH^+^ + He dissociation threshold corresponding to the zero of the relative energy.

**Figure 6:**
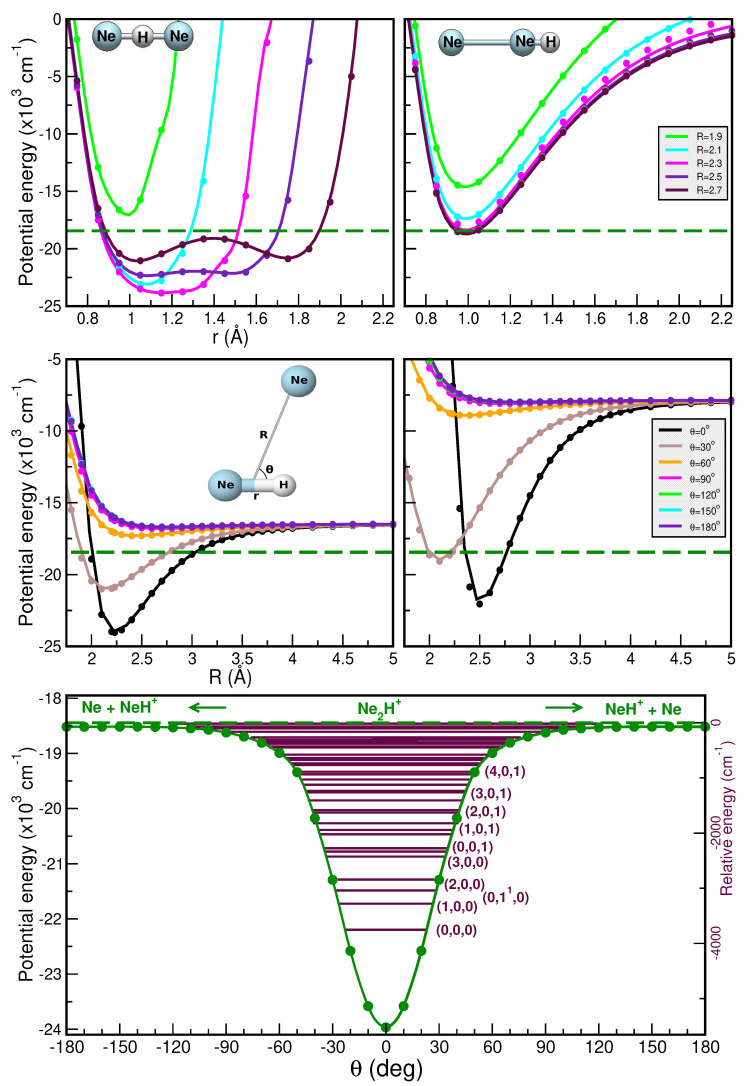
As in Figure 5 for the [Ne_2_H]^+^ molecule, for *r* = 1.15 Å (**left middle panel**) and 1.55 Å (**right middle panel**). The dashed green lines indicate the NeH^+^ + Ne dissociation threshold corresponding to the zero of the relative energy.

Thus, Figure 5 and Figure 6 present a comparison of the RKHS ML-PES (in solid lines) and the CCSD(T)/CBS[56] energies (in circle symbols) as a function of *r* (upper panels), *R* (middle panels), and θ (lower panels). In the upper panels we show the effect on the interaction of the shared proton motion between the two Ng atoms for the linear Ng–H^+^–Ng (upper left panels) and antilinear Ng–Ng–H^+^ (upper right panels) configurations of the [He_2_H]^+^ and [Ne_2_H]^+^ complexes, respectively, keeping fixed the *R* distances at the range of 1.5 to 2.7 Å. In the middle panels the potential curves are plotted for fixed θ from 0 to 180^∘^ for *r* = 0.90/1.15 Å (left panels) and 1.35/1.55 Å (right panels) as a function of *R*, while the minimum energy path (MEP) plots are shown in the lower panels as a function of θ optimizing both the *r* and *R* coordinates. As it is seen, the RKHS ML-PES smoothly reproduces the ab initio data for all analyzed cases. Additionally, the full 3D RKHS ML-potential for [Ng_2_H]^+^ cations are also presented using two-dimensional contour plots in Figure S2 (see Supplementary Material) in the (θ, *R*) and (*r*, *R*) planes, over from −24000 cm^−1^ to −15000 cm^−1^ for both cases.

In this manner, one can observe that the global minimum corresponds to the linear [Ng–H–Ng]^+^ configuration, while the local minimum coincides with the antilinear [Ng–Ng–H]^+^ configuration in both cases. For the [He_2_H]^+^ system, the well depth of the global minimum is equal to 21100.37 cm^−1^ in the RKHS ML-PES and is located at *R* = 1.69 Å and *r* = 0.95 Å, whereas the second minimum presents an energy of −16778.80 cm^−1^ with *R* = 2.27 Å. Regarding the [Ne_2_H]^+^ complex, the well depth is equal to 24001.01 cm^−1^ in the ML-PES and is located at *R* = 2.23 Å and *r* = 1.15 Å, whereas the local minimum presents an energy of −18728.80 cm^−1^ with *R* = 2.69 Å. Moreover, the potential energy barrier between the global and local potential minima is *R* = 2.27 Å with an energy of −16759.10 cm^−1^ for [He_2_H]^+^ and at *R* = 2.70 Å with an energy of −18724.95 cm^−1^ for [Ne_2_H]^+^.

### 2.3. Bound-State Quantum Calculations: Vibrational Spectral Bands Assignment

We employed the MCTDH method, as implemented in the MCTDH package of codes [81], to solve the time-dependent Schrödinger equation [82,83], $i\Psi^{J,K}=H_{K,K}^{J}\Psi^{J,K}+H_{K,K\pm 1}^{J}\Psi^{J,K\pm 1}$, where $\Psi^{J}\left(r,R,\theta ,\phi \right)=\frac{1}{\sqrt{2\pi }}\sum_{K}\Psi^{JK}\left(r,R,\theta \right)\exp^{iK\phi }$, and the Hamiltonian terms being,

(2) $$H_{K,K\pm 1}^{J}=-\frac{\sqrt{J(J+1)-K(K\pm 1)}}{2\mu_{1}R^{2}}\left(\mp \frac{\partial }{\partial \theta }-K\cot\theta \right)$$

(3) $$\begin{array}{cc} H_{K,K}^{J}= & -\frac{1}{2\mu_{1}}\frac{\partial^{2}}{\partial R^{2}}-\frac{1}{2\mu_{2}}\frac{\partial^{2}}{\partial r^{2}}-\frac{1}{2\mu_{2}r^{2}\sin\theta }\left(\frac{\partial }{\partial \theta }\sin\theta \frac{\partial }{\partial \theta }-\frac{K^{2}}{\sin\theta }\right)\\ \; & +\frac{1}{2\mu_{1}R^{2}}\left(J\left(J+1\right)-2K^{2}-\frac{1}{\sin\theta }\left(\frac{\partial }{\partial \theta }\sin\theta \frac{\partial }{\partial \theta }-\frac{K^{2}}{\sin\theta }\right)\right)+V\left(r,R,\theta \right) \end{array}$$

The (r,R,θ) Jacobi coordinates are chosen with the *R* being parallel to the *Z* body-fixed (BF) axis and the *r* coordinate lying in the XZ-plane. The azimuthal Euler angle ϕ specifies the orientation of the BF around *R*. The total angular momentum J=L+j is conserved, with *L* and *j* being the orbital and diatomic angular momentum operators, respectively, and its projection along *Z*, $J_{Z}$ = *K*, being the same as that of $j_{Z}$, as $l_{Z}$ = 0 by construction. μ1=mNg(mNg+m)HM and $\mu_{2}=\frac{m_{Ng}m_{H}}{m_{Ng}+m_{H}}$ are the reduced masses, with $M=2m_{Ng}+m_{H}$ the total mass of Ng_2_H^+^ and $m_{Ng}$, $m_{H}$ the mass of Ng=He or Ne and H or D atoms (isotopes), respectively.

The V(r,R,θ) term is the RKHS ML-PES for each Ng_2_H^+^ cation, and within the MCTDH method [84,85,86] the POTFIT algorithm [87] was employed to transform these terms as a sum of products of single-particle operators, namely natural potentials (NPs). While POTFIT is highly efficient for representing low-dimensional PESs, it is true that the accuracy of angular coupling terms may be affected if the decomposition is not sufficiently converged. In our case, convergence tests have shown that they are accurate enough to reliably describe all key features of the PESs. Thus, the contraction is constructed over the angular coordinate θ, and 25 NPs are used in the POTFIT calculations for each *r* and *R* coordinates. In these calculations, we considered the relevant region below the Ng+Ng+H^+^ dissociation threshold with a root mean square error (RMSE) of fit in this region smaller than 2.5 cm^−1^.

The wavefunction is also expanded in a sum of products of time-dependent basis, ψ(Q,t), namely single-particle functions (SPFs), as

(4) $$\Psi^{JK}=\sum_{nkl}A_{nkl}^{J}\left(t\right)\psi_{n}^{\left(1\right)}\left(r,t\right)\psi_{k}^{\left(2\right)}\left(R,t\right)\psi_{l}^{\left(3\right)}\left(\theta ,K,t\right),$$

with $A_{nkl}^{J}$ being the time-dependent coefficients and *Q* the nuclear coordinates. This makes MCTDH much more compact and memory-efficient, especially for strongly coupled or anharmonic systems, allowing it to handle multidimensional complex systems. Both the wavefunction and Hamiltonian operators are represented on a grid of the (r,R,θ) coordinates ranging in the configuration space of interest, with the variable *K* taking only integer values. We have run several convergence tests, and we have found that convergence is achieved using 51 harmonic oscillator (HO) discrete variable representation (DVR) functions in each *r* and *R* coordinates in the range of [0.6, 1.95]/[0.7, 2.25] and [1.35, 3.5]/[1.8, 4.0] Å, respectively, while for the angular coordinate θ we used 55 Leg DVR functions in the [0, 2π] interval.

The improved relaxation (IR) and the block improved relaxation (BIR) methods [88,89], as implemented in the MCTDH package [81], were used to calculate the ground and excited rovibrational states. We have used a set of (18, 18, 18) SPFs in each coordinate in the IR and BIR MCTDH calculations, and we calculated a total of up to 20 and 50/70 rotational-vibrational states considering the H and D isotopes in the He_2_H^+^/D^+^ and Ne_2_H^+^/D^+^, respectively. The total propagation time was 312 fs for achieving a convergence of less than 10^−4^ cm^−1^. All calculated *J* = 0, 1 and 2 states are given in the Supplementary Material, while all vibrational energy levels and their assignment (when possible) are listed in Table 1 and Table 2 and also shown in Figure 5 and Figure 6 for the He_2_H^+^ and Ne_2_H^+^, respectively. Comparisons with values reported in previously reported theoretical studies [34,35,37] are included in Table 1 and Table 2.

The anharmonic ZPEs are 2310.5/1802.55 and 1933.1/1384.65 cm^−1^ for He_2_H^+^/Ne_2_H^+^ and He_2_D^+^/Ne_2_D^+^, respectively, with deuteration reducing the ZPE values by 377.8/417.9 cm^−1^. By comparing with the ZPEs previously reported, we found differences of 29 and 143 cm^−1^ for He_2_H^+^, 85 and 172 cm^−1^ for He_2_D^+^ [34,37], and 34 cm^−1^ [35] for Ne_2_H^+^. One can also observe that the present ML-PESs predict 5/10 bound vibrationally excited levels for He_2_H^+^/He_2_D^+^, with energies that are significantly different from previous results available for He_2_H^+^ [34,37]. In turn, 34/70 bound vibrationally excited levels are obtained for Ne_2_H^+^/Ne_2_D^+^, whose energies are in better accord with the recently reported 22 ones [35] for Ne_2_H^+^.

**Figure 7:**
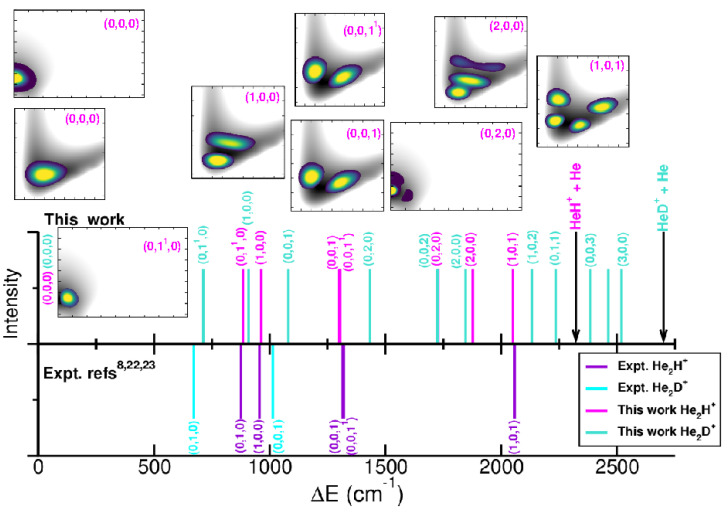
Comparison of the calculated and experimental [8,22,23] rovibrational transitions for the He_2_H^+^.

Figure 7 and Table 2 display 2D probability distributions of various rovibrational $\nu_{1},\nu_{2}^{J},\nu_{3}$ states in the (*r*, *R*) or (θ, *R*) or (θ, *r*) planes, where $\nu_{1}$ is the anharmonic symmetric Ng–H^+^ stretch, $\nu_{2}$ the anharmonic doubly degenerate proton bend vibration, and $\nu_{3}$ the anharmonic antisymmetric Ng–H^+^ stretching or shared-proton mode. The assignment of each energy level has been extracted by analyzing the corresponding nodal structure of the probability density functions (see corresponding plots in Figure 7 and Table 2). As shown, the ground vibrational state (0,0,0) has zero nodes, the (1,0,0) has one node in *R*, the (0,0,1) shows one node in *r*, while the (1,0,1) has one node in each *r* and *R* coordinate. In turn, the (0,1^1^,0) and (0,2,0) density functions exhibit bending excitations with one and two nodes in the θ coordinate, respectively.

Regarding the $\nu_{1}$ and $\nu_{3}$ bands, our results predict values of 962.1 and 1300.4 cm^−1^ for He_2_H^+^, with $\nu_{1}$ closely matching previously reported theoretical values of 963.6 and 954.2 cm^−1^ [34,37], while the $\nu_{2}$ is found at 886.7 cm^−1^. A comparison with recent experimental data [8,22,23] for specific rovibrational bands of He_2_H^+^ is presented in Figure 7. Notably, the $\nu_{3}$ band has been observed at 1315.8444 cm^−1^, along with seven of its rovibrational lines [8], including the (0,0,1^1^) transition at 1320.1882 cm^−1^. Additional estimates reported the $\nu_{2}$ bending mode at 874.9 cm^−1^ or the $\nu_{1}$ at 956.6 cm^−1^ or the combination band $\nu_{1}+\nu_{3}$ at 2057.9 cm^−1^ [8,22,23]; for reference, the $\nu_{2}$ and $\nu_{3}$ fundamentals have been estimated at 673 and 1014 cm^−1^ for He_2_D^+^ [14]. Our predictions underestimate the $\nu_{3}$ and the $\nu_{1}+\nu_{3}$ band by 15.4 and 7.1 cm^−1^, respectively, while overestimating the $\nu_{1}$ and $\nu_{2}$ by 11.8 and 5.5 cm^−1^, respectively. In the case of He_2_D^+^ our calculations predicted the three fundamental bands at 907.6, 717.9 and 1080.4 cm^−1^.

In turn, for Ne_2_H^+^ the $\nu_{1}$ and $\nu_{3}$ vibrational bands are found at 475.5 and 1418.3 cm^−1^, with the $\nu_{3}$ band comparing well to the reported value of 1432 cm^−1^ [39]. Additionally, the $\nu_{1}+\nu_{3}$, 2$\nu_{1}+\nu_{3}$ and 3$\nu_{1}+\nu_{3}$ combination bands are predicted at 1807.3, 2163.8 and 2485.3 cm^−1^, respectively, in close agreement with the 1814, 2182 and 2541 cm^−1^ values obtained from a coupled anharmonic model [39]. To date no experimental measurements have been reported for the Ne_2_H^+^/Ne_2_D^+^ species, and thus the present predictions await experimental verification.

## 3. Conclusions

The present study focuses on the computational spectroscopic characterization of the proton-bound light noble-gas Ng_2_H^+^ cations. Our results were obtained from high-level and well-converged ab initio electronic structure calculations, and newly trained RKHS ML-PESs were developed using high-quality CCSD(T)/CSB[56] set of data, followed by nuclear quantum bound state computations within the MCTDH framework employing the ML-PESs. Rovibrational states were calculated and assignments for several vibrational bands were provided. Comparisons of the zero-point energies, fundamental vibrational frequencies, and combination bands with previously reported theoretical estimates and recent experimental measurements on He_2_H^+^ were also presented and discussed. We have found that vibrational fundamentals bands are in close agreement with the experimental values reported for He_2_H^+^, while our results for Ne_2_H^+^ provide detailed and precise spectroscopic predictions that may serve as benchmarks for future laboratory or astrophysical detections.

Looking ahead, the quantum methodologies and insights developed in this work can be extended to the heavier proton-bound noble gas complexes, broadening the exploration of spectral features of this intriguing class of molecular ions. The success of the ML-PES strategy also opens avenues for modeling larger and more complex weakly bound systems, bridging the gap between computational predictions and experimental efforts, an ongoing challenge for computational spectroscopy. We anticipate that the present results will stimulate further experimental studies and will contribute to a deeper understanding of such noble gas cations in the ISM.

## Figures and Tables

**Table 1:** Vibrational energies (in cm^−1^) and their ($\nu_{1}$, $\nu_{2}$, $\nu_{3}$) assignment for the [^4^He_2_H]^+^ and [^4^He_2_D]^+^ bound states from the MCTDH calculations using the CCSD(T)/CBS[56] RKHS PES with the zero in energy at the He + HeH^+^ dissociation. Comparison with previous studies [34,37] is also shown.

	[He24${\mathrm{H]}}^{+}$		[He24${\mathrm{D]}}^{+}$	
	**This Work**	**From Ref. [37]/Ref. [34]**	**This Work**	**From Ref. [37]/Ref. [34]**
v/D_*e*_	−4633.10	−4603.92/−4661.88	–	–
0	−2322.6 (0,0,0)	−2321.77 (0,0,0)/−2493.87	−2700.74 (0,0,0)	−2755.92 (0,0,0)/−2900.37
1	−1360.5 (1,0,0)	−1358.16 (1,0,0)/−1539.712	−1793.2 (1,0,0)	−1775.16 (1,0,0)/−1964.77
2	−1022.2 (0,0,1)	−564.18 (2,0,0)/−1133.21	−1620.4 (0,0,1)	−1384.04 (0,2,0)/−1822.81
3	−594.8 (0,2,0)	−462.16 (0,2,0)/−821.88	−1267.8 (0,2,0)	−916.32 (2,0,0)/−1630.05
4	−446.4 (2,0,0)	−422.16 (1,2,0)/−623.47	−976.8 (0,0,2)	−609.23 (1,2,0)/−1147.73
5	−271.8 (1,0,1)	+134.32/−367.79	−855.9 (2,0,0)	–/−958.19
6	+84.6 (2,0,1)	–/−84.69	−567.5 (1,0,2)	–/−850.11
7	+290.2 (1,1,1)	–	−464.8 (0,1,1)	–/−779.13
8	+348.3	–	−315.1 (0,0,3)	–/−537.97
9	+426.3	–	−239.1	–/−453.28
10	–	–	−182.7 (3,0,0)	–/−329.88
11	–	–	+45.8 (0,2,1)	–/−278.26
12	–	–	+138.0 (1,0,1)	–/−145.99
13	–	–	+166.5	–/−18.55

**Table 2:** Same as Table 1 for the [^20^Ne_2_H]^+^ and [^20^He_2_D]^+^ isotopologues calculated with the corresponding CSD(T)/CBS[56] RKHS ML-PES, and comparison with previous studies [35], considering the energy of Ne + NeH^+^ asymptote as zero.

	[Ne220H]+			[Ne220${\mathrm{D]}}^{+}$
	**This Work**		**From Ref. [35] ^*a/b*^**	**This Work**
v/D_*e*_	−5556.29	2D plots	−5807.96	–
0	−3753.3 (0,0,0)	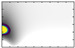	−3971.47	−4170.5 (0,0,0)
1	−3277.8 (1,0,0)	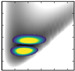	−3514.96	−3697.2 (1,0,0)
2	−2841.8 (2,0,0)	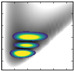	−3068.13	−3260.8 (2,0,0)
3	−2426.7 (3,0,0)	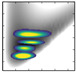	−2634.21	−3119.9 (0,0,1)
4	−2335.0 (0,0,1)	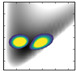	−2535.81	−3078.9 (0,2,0)
5	−2273.6 (0,2,0)	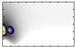	−2444.67	−2843.1 (3,0,0)
6	−2022.5 (4,0,0)	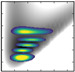	−2213.99	−2693.1 (1,0,1)
7	−1946.0 (1,0,1)	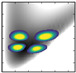	−2152.69	−2631.3 (1,1,0)
8	−1826.9 (1,1,0)	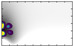	−2005.90	−2435.6 (4,0,0)
9	−1632.0 (5,0,0)	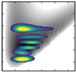	−1809.10	−2322.8 (2,0,1)
10	−1589.5 (2,0,1)	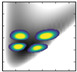	−1784.91	−2217.0 (2,1,0)
11	−1414.3 (2,1,0)	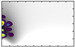	−1585.69/−1586.49	−2185.5 (0,1,1)
12	−1268.0 (3,0,1)	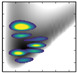	−1434.86/−1435.67	−2056.3 (5,0,0)
13	−1243.4 (6,0,1)	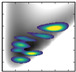	−1420.34/−1422.76	−1977.9
14	−1143.5 (1,0,1)	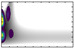	−1292.10/−1294.52	−1966.8 (4,0,1)
15	−1039.7 (3,1,0)	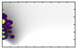	−1200.96/−1204.19	−1940.6 (0,3,0)
16	−942.5 (4,0,1)	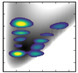	−1096.91/−1104.98	−1814.2
17	−898.6		−1051.75/−1063.04	−1779.4 (1,1,1)
18	−819.9		−993.68/−995.29	−1701.8
19	−766.6		−926.73/−933.99	−1631.0 (4,0,1)
20	−734.7		−903.34/−905.76	−1584.5
21	−674.5		−821.07/−831.56	−1521.1
22	−639.2			−1449.2
…				…
34	−16.0			−897.2
…				…
70				−7.0

The superscript a/b in Table 2 corresponds to the DVR3D/TDWP results given in ref. [35].

## Data Availability

The data that support the findings of this study are available within the article as well as from the authors upon reasonable request.

## Supplementary Materials

The following supporting information can be downloaded at: https://www.mdpi.com/article/10.3390/molecules30112440/s1 Table S1. Calculated vibrational normal modes of the global minimum (in cm^−1^) CCSD(T)/AV6Z level of theory for ^4^He-H-^4^He and ^20^Ne-H-^20^Ne conformers. *MP2/AV6Z vibrational intensities in km/mol are in parenthesis. The vibrational normal modes where the intensities are zero by symmetry have no values; Table S2. Calculated relative dissociation/formation energies (in cm^−1^) CCSD(T)/AV6Z level of theory for [He_2_H]^+^ and [Ne_2_H]^+^, taking to account the energy of these systems like zero; Table S3. Formation energies in kcal/mol for NgH^+^-Ng (where Ng is He and Ne atoms) at its linear configurations using CCSD(T)/CBS[56] level of theory. Their comparison with theoretical values from previous studies is also presented; Figure S1. Histograms of different size training data set employed in building up the RHKHS ML-PES models for [He_2_H]^+^ (left blue panels) and [Ne_2_H]^+^ (riht green panels) complexes; Figure S2. Contour plots of the He_2_H^+^ (upper panels) and Ne_2_H^+^ (lower panels) RKHS ML-PESs in the (r,R) (left panels) and (θ,R)-planes (right panels) with θ = 0°, and *r* = 0.95 and 1.15 Å respectively. Potential energy values are given in cm^−1^; ‘SI-energiesJ=0-1-2.ods’ file includes *J* = 0, 1 and 2 energy levels of the [He_2_H]^+^ and [Ne_2_H]^+^ complexes.

## References

[1] Tennyson J. (2011). Accurate variational calculations for line lists to model the vibrational-rotation spectra of hot astrophysical atmospheres. WIREs Comput. Mol. Sci..

[2] Yu Q., Bowman J.M., Fortenberry R.C., Mancini J.S., Lee T.J., Crawford T.D., Klemperer W., Francisco J.S. (2015). Structure, Anharmonic Vibrational Frequencies, and Intensities of NNHNN^+^. J. Phys. Chem. A.

[3] Simmons J., Carrington T. (2023). Computing vibrational spectra using a new collocation method with a pruned basis and more points than basis functions: Avoiding quadrature. J. Chem. Phys..

[4] Valdés Á., Prosmiti R. (2014). First-principles simulations of vibrational states and spectra for $H_{5}^{+}$ and $D_{5}^{+}$ clusters using multiconfiguration time-dependent Hartree approach. Spectrochim. Acta A.

[5] Császár A.G., Fábri C., Szidarovszky T., Mátyus E., Furtenbacher T., Czakó G. (2012). The fourth age of quantum chemistry: Molecules in motion. Phys. Chem. Chem. Phys..

[6] Carrillo-Bohórquez O., Valdés Á., Prosmiti R. (2023). Computational Energy Spectra of the H2O@C70 Endofullerene. ChemPhysChem.

[7] Valdés Á., Cabrera-Ramírez A., Prosmiti R. (2023). Confining CO_2_ inside sI clathrate-hydrates: The impact of the CO_2_-wate interaction on quantized dynamics. J. Comput. Chem..

[8] Salomon T., Baddeliyanage C., Schladt C., Simkó I., Császár A.G., Silva W.G.D.P., Schlemmer S., Asvany O. (2025). High-resolution leak-out spectroscopy of ${\mathrm{HHe}}_{2}^{+}$. Phys. Chem. Chem. Phys..

[9] (2022). Molecules in Space. https://cdms.astro.uni-koeln.de/classic/molecules.

[10] Güsten R., Wiesemeyer H., Neufeld D., Menten K., Graf U., Jacobs K., Klein B., Ricken O., Risacher C., Stutzki J. (2019). Astrophysical detection of the helium hydride ion HeH^+^. Nature.

[11] Barlow M., Swinyard B., Owen P., Cernicharo J., Gomez H., Ivison R., Krause O., Lim T., Matsuura M., Miller S. (2013). Detection of a Noble Gas Molecular Ion, ^36^ArH^+^, in the Crab Nebula. Science.

[12] Müller H., Muller S., Schilke P., Bergin E., Black J., Gerin M., Lis D., Neufeld D., Suri S. (2015). Detection of extragalactic argonium, ArH^+^, toward PKS 1830-211. A&A.

[13] (2015). NASA’s LADEE Spacecraft Finds Neon in Lunar Atmosphere. https://sservi.nasa.gov/articles/nasas-ladee-spacecraft-finds-neon-in-lunar-atmosphere/.

[14] Asvany O., Schlemmer S., Szidarovszky T., Császár A.G. (2019). Infrared Signatures of the ${\mathrm{HHe}}_{n}^{+}$ and ${\mathrm{DHe}}_{n}^{+}$ (n = 3–6) Complexes. J. Phys. Chem. Lett..

[15] Kojima T.M., Kobayashi N., Kaneko Y. (1992). Formation of helium cluster ions HHe_x_^+^ (x ≤ 14) and H_3_He_x_^+^ (x ≤ 13) in a very low temperature drift tube. Z. Phys. D-Atoms. Mol. Clust..

[16] Bartl P., Leidlmair C., Denifl S., Scheier P., Echt O. (2013). Cationic Complexes of Hydrogen with Helium. Chem. Phys. Chem.

[17] Lundberg L., Bartl P., Leidlmair C., Scheier P., Gatchell M. (2020). Protonated and Cationic Helium Clusters. Molecules.

[18] Apkarian V.A., Schwentner N. (1999). Molecular Photodynamics in Rare Gas Solids. Chem. Rev..

[19] Beyer M., Lammers A., Savchenko E.V., Niedner-Schatteburg G., Bondybey V.E. (1999). Proton solvated by noble-gas atoms: Simplest case of a solvated ion. Phys. Chem. Chem. Phys..

[20] Fridgen T.D., Parnis J.M. (1998). Electron bombardment matrix isolation of Rg/Rg/methanol mixtures (Rg = Ar, Kr, Xe): Fourier-transform infrared characterization of the proton-bound dimers Kr_2_H^+^, Xe_2_H^+^, (ArHKr)^+^ and (ArHXe)^+^ in Ar matrices and (KrHXe)^+^ and Xe_2_H^+^ in Kr matrices. J. Chem. Phys..

[21] Kunttu H., Seetula J., Räsänen M., Apkarian V.A. (1992). Photogeneration of ions via delocalized charge transfer states. I. Xe_2_H^+^ and Xe_2_D^+^ in solid Xe. J. Chem. Phys..

[22] Tøpfer M., Jensen A., Nagamori K., Kohguchi H., Szidarovszky T., Császár A.G., Schlemmer S., Asvany O. (2020). Spectroscopic signatures of ${\mathrm{HHe}}_{2}^{+}$ and ${\mathrm{HHe}}_{3}^{+}$. Phys. Chem. Chem. Phys..

[23] Simkó I., Schran C., Brieuc F., Fábri C., Asvany O., Schlemmer S., Marx D., Császár A.G. (2023). Quantum Nuclear Delocalization and its Rovibrational Fingerprints. Angew. Chem. Int. Ed..

[24] Poshusta R.D., Haugen J.A., Zetik D.F. (1969). Ab Initio Predictions for Very Small Ions. J. Chem. Phys..

[25] Poshusta R.D., Siems W.F. (1971). Ab Initio Calculations on He_2_H^+^. J. Chem. Phys..

[26] Milleur M.B., Matcha R.L., Hayes E.F. (1974). Theoretical studies of hydrogen-rare gas complexes: He_n_H and He_n_H^+^ clusters. J. Chem. Phys..

[27] Dykstra C.E. (1983). The strong hydrogen bond in HeHHe^+^ and its weak counterpart in ${\mathrm{HeH}}_{3}^{+}$. J. Mol. Struct. Theochem.

[28] Baccarelli I., Gianturco F.A., Schneider F. (1997). Stability and fragmentation of protonated helium dimers from ab initio calculations of their potential energy surfaces. J. Phys. Chem. A.

[29] Kim S.T., Lee J.S. (1999). Ab initio study of He_2_H^+^ and Ne_2_H^+^: Accurate structure and energetics. J. Chem. Phys..

[30] Filippone F., Gianturco F.A. (1998). Charged chromophoric units in protonated rare-gas clusters: A dynamical simulation. Eur. Phys. Lett..

[31] Fortenberry R.C. (2017). Rovibrational Characterization and Interstellar Implications of the Proton-Bound, Noble Gas Complexes: ArHAr^+^, NeHNe^+^, and ArHNe^+^. ACS Earth Space Chem..

[32] Stephan C.J., Fortenberry R.C. (2017). The interstellar formation and spectra of the noble gas, proton-bound HeHHe^+^, HeHNe^+^ and HeHAr^+^ complexes. Mon. Notices Royal Astron. Soc..

[33] Fortenberry R.C., Wiesenfeld L. (2020). A Molecular Candle Where Few Molecules Shine: HeHHe^+^. Molecules.

[34] Panda A., Sathyamurthy N. (2003). Bound and Quasibound States of He_2_H^+^ and He_2_D^+^. J. Phys. Chem. A.

[35] Koner D., Barrios Herrera L., Gonzalez-Lezana T., Panda A. (2016). Scattering study of the Ne + NeH^+^(v = 0, j = 0) → NeH^+^ + Ne reaction on an ab initio based analytical potential energy surface. J. Chem. Phys..

[36] Császár A.G., Tamás Szidarovszky O.A., Schlemmer S. (2019). Fingerprints of microscopic superfluidity in ${\mathrm{HHe}}_{n}^{+}$ clusters. Mol. Phys..

[37] Lee J.S., Secrest D. (1986). A calculation of the rotation–vibration states of He_2_H^+^. J. Chem. Phys..

[38] Liang J.J., Yang C.L., Wang L.Z., Zhang Q.G. (2012). A new analytical potential energy surface for the singlet state of He_2_H^+^. J. Chem. Phys..

[39] Tan J.A., Kuo J.L. (2019). A theoretical study on the infrared signatures of proton-bound rare gas dimers (Rg–H^+^–Rg), Rg=Ne, Ar, Kr, and Xe. J. Chem. Phys..

[40] Murrell J.N. (1984). Molecular Potential Energy Functions.

[41] Polyansky O.L., Prosmiti R., Klopper W., Tennyson J. (2000). An accurate, global, ab initio potential energy surface for the $H_{3}^{+}$ molecule. Mol. Phys..

[42] Prosmiti R., Buchachenko A.A., Villarreal P., Delgado-Barrio G. (2001). Modeling the $H_{5}^{+}$ potential-energy surface: A first attempt. Theor. Chem. Acc..

[43] Huang X., Braams B., Bowman J., Kelly R., Tennyson J., Groenenboom G., van der Avoird A. (2008). New ab initio potential energy surface and the vibration-rotation-tunneling levels of (H_2_O)_2_ and (D_2_O)_2_. J. Chem. Phys..

[44] Braams B., Bowman J. (2009). Permutationally invariant potential energy surfaces in high dimensionality. Int. Rev. Phys. Chem..

[45] Aguado A., Barragán P., Prosmiti R., Villarreal G.D.B.P., Roncero O. (2010). A new accurate and full dimensional potential energy surface of $H_{5}^{+}$ based on a triatomics-in-molecules analytic functional form. J. Chem. Phys..

[46] Barragán P., Prosmiti R., Wang Y., Bowman J. (2012). Full-dimensional (15-dimensional) ab initio analytical potential energy surface for the $H_{7}^{+}$ cluster. J. Chem. Phys..

[47] Arismendi-Arrieta D., Riera M., Bajaj P., Prosmiti R., Paesani F. (2016). i-TTM Model for Ab Initio-Based Ion-Water Interaction Potentials. 1. Halide-Water Potential Energy Functions. J. Phys. Chem. B.

[48] Goncalves C., Galvão B.R., Mota V., Braga J., Varandas A. (2018). Accurate Explicit-Correlation-MRCI-Based DMBE Potential-Energy Surface for Ground-State CNO. J. Phys. Chem. A.

[49] Behler J., Parrinello M. (2007). Generalized Neural-Network Representation of High-Dimensional Potential-Energy Surfaces. Phys. Rev. Lett..

[50] Behler J. (2016). Perspective: Machine learning potentials for atomistic simulations. J. Chem. Phys..

[51] Manzhos S., Carrington T.J. (2021). Neural Network Potential Energy Surfaces for Small Molecules and Reactions. Chem. Rev..

[52] Unke O.T., Koner D., Patra S., Käser S., Meuwly M. (2020). High-dimensional potential energy surfaces for molecular simulations: From empiricism to machine learning. Mach. Learn. Sci. Technol..

[53] Noe F., Tkatchenko A., Muller K.R., Clementi C. (2020). Machine Learning for Molecular Simulation. Annu. Rev. Phys. Chem..

[54] Dral P. (2020). Quantum Chemistry in the Age of Machine Learning. J. Phys. Chem. Lett..

[55] Gao X., Ramezanghorbani F., Isayev O., Smith J., Roitberg A. (2020). TorchANI: A Free and Open Source PyTorch-Based Deep Learning Implementation of the ANI Neural Network Potentials. J. Chem. Inf. Model..

[56] Unke O.T., Chmiela S., Sauceda H.E., Gastegger M., Poltavsky I., Schütt K.T., Tkatchenko A., Müller K.R. (2021). Machine Learning Force Fields. Chem. Rev..

[57] Pinheiro M., Ge F., Ferré N., Dral P.O., Barbatti M. (2021). Choosing the right molecular machine learning potential. Chem. Sci..

[58] Montes de Oca-Estévez M.J., Prosmiti R. (2023). Quantum computations in heavy noble-gas hydride cations: Reference energies and new spectroscopic data. J. Mol. Graph. Model..

[59] Montes de Oca-Estévez M.J., Valdés Á., Prosmiti R. (2024). A kernel-based machine learning potential and quantum vibrational state analysis of the cationic Ar hydride (Ar_2_H^+^). Phys. Chem. Chem. Phys..

[60] Montes de Oca-Estévez M.J., Valdés Á., Koner D., González-Lezana T., Prosmiti R. (2024). Quantum computations on a new neural network potential for the proton-bound noble-gas Ar_2_H^+^ complex: Isotopic effects. Chem. Phys. Letts..

[61] Montes de Oca-Estévez M.J., Prosmiti R. (2024). Microsolvation of a Proton by Ar Atoms: Structures and Energetics of Ar_n_H^+^ Clusters. Molecules.

[62] Dral P. (2019). MLatom: A program package for quantum chemical research assisted by machine learning. J. Comput. Chem..

[63] Abbott A., Turney J., Zhang B., Smith D., Altarawy D., Schaefer H. (2019). PES-Learn: An Open-Source Software Package for the Automated Generation of Machine Learning Models of Molecular Potential Energy Surfaces. J. Chem. Theory Comput..

[64] Unke O., Meuwly M. (2019). PhysNet: A Neural Network for Predicting Energies, Forces, Dipole Moments, and Partial Charges. J. Chem. Theory Comput..

[65] Chmiela S., Sauceda H., Poltavsky I., Müller K.R., Tkatchenko A. (2019). sGDML: Constructing accurate and data efficient molecular force fields using machine learning. Comput. Phys. Commun..

[66] Shao Y., Hellstrom M., Mitev P.D., Knijff L., Zhang C. (2020). PiNN: A Python Library for Building Atomic Neural Networks of Molecules and Materials. J. Chem. Inf. Model..

[67] Ho T., Rabitz H. (1996). A general method for constructing multidimensional molecular potential energy surfaces from ab initio calculations. J. Chem. Phys..

[68] Hollebeek T., Ho T.S., Rabitz H. (1997). A fast algorithm for evaluating multidimensional potential energy surfaces. J. Chem. Phys..

[69] Hollebeek T., Ho T.S., Rabitz H. (1999). Constructing multidimensional molecular potential energy surfaces from ab initio data. Ann. Rev. Phys. Chem..

[70] Ho T., Rabitz H. (2000). Proper construction of ab initio global potential surfaces with accurate long-range interactions. J. Chem. Phys..

[71] Ho T., Rabitz H. (2003). Reproducing kernel Hilbert space interpolation methods as a paradigm of high dimensional model representations: Application to multidimensional potential energy surface construction. J. Chem. Phys..

[72] Delgado-Tellez L., Valdés A., Prosmiti R., Villarreal P., Delgado-Barrio G. (2012). HeI_2_ interaction potential based on an interpolation scheme. Int. J. Quantum Chem..

[73] Kalemos A., Valdés A., Prosmiti R. (2012). Theoretical investigation of the HeI_2_(E^3^*Π*_g_) ion-pair state: Ab initio intermolecular potential and vibrational levels. J. Chem. Phys..

[74] Alharzali N., Berriche H., Villarreal P., Prosmiti R. (2019). Theoretical Study of Cationic Alkali Dimers Interacting with He: ${\mathrm{Li}}_{2}^{+}$-He and ${\mathrm{Na}}_{2}^{+}$-He van der Waals Complexes. J. Phys. Chem. A.

[75] Alharzali N., Rodríguez-Segundo R., Prosmiti R. (2021). Modelling interactions of cationic dimers in He droplets: Microsolvation trends in He_n_$K_{2}^{+}$ clusters. Phys. Chem. Chem. Phys..

[76] Werner H.J., Knowles P.J., Celani P., Györffy W., Hesselmann A., Kats D., Knizia G., Köhn A., Korona T., Kreplin D. MOLPRO, Version 2012.1, a Package of Ab Initio Programs. http://www.molpro.net.

[77] (2020). DENEB 1.30 Beta: The Nanotechnology Software by Atelgraphics. https://sites.google.com/view/atelgraphics/atelgraphics/.

[78] Montes de Oca-Estévez M.J., Prosmiti R. (2021). Computational Characterization of Astrophysical Species: The Case of Noble Gas Hydride Cations. Front. Chem..

[79] Grabowski S.J., Ugalde J.M., Andrada D.M., Frenking G. (2016). Comparison of Hydrogen and Gold Bonding in [XHX]^−^, [XAuX]^−^, and Isoelectronic [NgHNg]^+^, [NgAuNg]^+^ (X=Halogen, Ng=Noble Gas). Eur. J. Chem..

[80] Schwartz C. (1962). Importance of Angular Correlations between Atomic Electrons. Phys. Rev..

[81] Worth G.A., Beck M.H., Jäckle A., Meyer H.D. (2007). The MCTDH Package, Version 8.2, (2000). H.-D. Meyer, Version 8.3 (2002),
Version 8.4 (2007). http://mctdh.uni-hd.de.

[82] Zhang J.Z.H. (1999). Theory and Application of Quantum Molecular Dynamics.

[83] Sukiasyan S., Meyer H.D. (2001). On the Effect of Initial Rotation on Reactivity. A Multi-Configuration Time-Dependent Hartree (MCTDH) Wave Packet Propagation Study on the H + D_2_ and D + H_2_ Reactive Scattering Systems. J. Phys. Chem. A.

[84] Meyer H.D., Manthe U., Cederbaum L.S. (1990). The Multi-Configurational Time-Dependent Hartree Approach. Chem. Phys. Lett..

[85] Beck M.H., Jäckle A., Worth G.A., Meyer H.D. (2000). The Multiconfiguration Time-Dependent Hartree Method: A Highly Efficient Algorithm for Propagating Wavepackets. Phys. Rep..

[86] Meyer H.D. (2012). Studying Molecular Quantum Dynamics with the Multiconfiguration Time-Dependent Hartree Method. WIREs Comput. Mol. Sci..

[87] Jäckle A., Meyer H.D. (1998). Product representation of potential energy surfaces. II. J. Chem. Phys..

[88] Meyer H.D., Quéré F.L., Léonard C., Gatti F. (2006). Calculation and Selective Population of Vibrational Levels with the Multiconfiguration Time-Dependent Hartree (MCTDH) Algorithm. Chem. Phys..

[89] Doriol L.J., Gatti F., Iung C., Meyer H.D. (2008). Computation of Vibrational Energy Levels and Eigenstates of Fluoroform Using the Multiconfiguration Time-Dependent Hartree Method. J. Chem. Phys..

