# Supplementary material for: Quantum Data-Driven Modeling of Interactions and Vibrational Spectral Bands in Cationic Light Noble-Gas Hydrides: [He2H]+ and [Ne2H]+
Source: Molecules. 2025 Jun 3;30(11):2440. doi: 10.3390/molecules30112440 (PMC12156169; doi:10.3390/molecules30112440)
Supplement: Supplementary file 1 [file molecules-30-02440-s001.zip › SI-material-2.pdf]

Quantum data-driven modeling of interactions and vibrational spectral bands in cationic light noble-gas hydrides: $[He_2H]^+$ and $[Ne_2H]^+$

María Judit Montes de Oca-Estévez[1], Álvaro Valdés [2,], and Rita Prosmiti[1*]

[1] *Institute of Fundamental Physics (IFF-CSIC), CSIC, Serrano 123, 28006 Madrid, Spain*

[2] *Departamento de Física, Universidad Nacional de Colombia, Sede Medellín, A. A., Medellín* 3840, *Colombia*

E-mail: *rita@iff.csic.es;Phone:+34-91-5616800Ext.442292

SUPPLEMENTARY MATERIAL

Table S1: Calculated vibrational normal modes of the global minimum (in cm^{-1}) CCSD(T)/AV6Z level of theory for ^{4}He-H-^{4}He and ^{20}Ne-H-^{20}Ne conformers.*MP2/AV6Z vibrational intensities in km/mol are in parenthesis. The vibrational normal modes where the intensities are zero by symmetry have no values.

	He_2H^+	Ne_2H^+
v_1 **(Symmetric NgH^+ stretch)**	1138.39	508.45
v_2 **(Degenerate proton bend)**	952.00(224.48)	752.91(253.42)
v_3 **(Asymmetric NgH^+ stretch)**	1547.74(2151.41)	1550.50(2507.76)

Table S2: Calculated relative dissociation/formation energies (in cm^{-1}) CCSD(T)/AV6Z level of theory for $[He_2H]^+$ and $[Ne_2H]^+$, taking to account the energy of these systems like zero.

**Reaction**	$[He_2H]^+$	$[Ne_2H]^+$
**Partial dissociation**		
$[Ng_2H]^+ \rightarrow NgH^+ + Ng$	4633.10	5556.29
$[Ng_2H]^+ \rightarrow H^+ + Ng_2$	21076.01	25688.88
$[Ng_2H]^+ \rightarrow H + Ng_2^+$	89750.39	76929.25
$[Ng_2H]^+ \rightarrow NgH + Ng^+$	109687.23	88560.08
**Total dissociation**		
$[Ng_2H]^+ \rightarrow H^+ + 2Ng$	22713.19	24041.34
$[Ng_2H]^+ \rightarrow H + Ng + Ng^+$	109702.64	88571.92

Table S3: Formation energies in kcal/mol for NgH$^+$-Ng (where Ng is He and Ne atoms) at its linear configurations using CCSD(T)/CBS[56] level of theory. Their comparison with theoretical values from previous studies is also presented.

	**First-step**	**Second-step**	**Overall Formation**
	$\mathbf{He}_{(g)}+\mathbf{H}^+_{(g)} \rightarrow [\mathbf{HeH}]^+_{(g)}$	$[\mathbf{HeH}]^+_{(g)}+\mathbf{He}_{(g)} \rightarrow [\mathbf{He_2H}]^+_{(g)}$	$\mathbf{2He}_{(g)}+\mathbf{H}^+_{(g)} \rightarrow [\mathbf{He_2H}]^+_{(g)}$
CCSD(T)/AV5Z	-47.05	-13.27	-60.32
CCSD(T)/AV6Z	-47.05	-13.25	-60.30
CCSD(T)/CBS[56]	-47.05	-13.01	-60.06
Ref.[32]	-	-13.20	-60.30
Ref.[33]	-	-11.30	-53.73
Ref.[28]	-	-11.99	-
Ref.[37]	-	-13.24	-
Ref.[34]	-	-13.33	-
Ref.[38]	-	-13.31	-
	$\mathbf{Ne}_{(g)}+\mathbf{H}^+_{(g)} \rightarrow [\mathbf{NeH}]^+_{(g)}$	$[\mathbf{NeH}]^+_{(g)}+\mathbf{Ne}_{(g)} \rightarrow [\mathbf{Ne_2H}]^+_{(g)}$	$\mathbf{2Ne}_{(g)}+\mathbf{H}^+_{(g)} \rightarrow [\mathbf{Ne_2H}]^+_{(g)}$
CCSD(T)/AV5Z	-51.51	-17.71	-69.21
CCSD(T)/AV6Z	-52.02	-16.97	-68.99
CCSD(T)/CBS[56]	-52.47	-15.77	-68.24
Ref.[40]	-52.14	-15.43	-67.58
Ref.[39]	-52.71	-15.55	-68.26
Ref.[35]	-53.43	-	-70.04

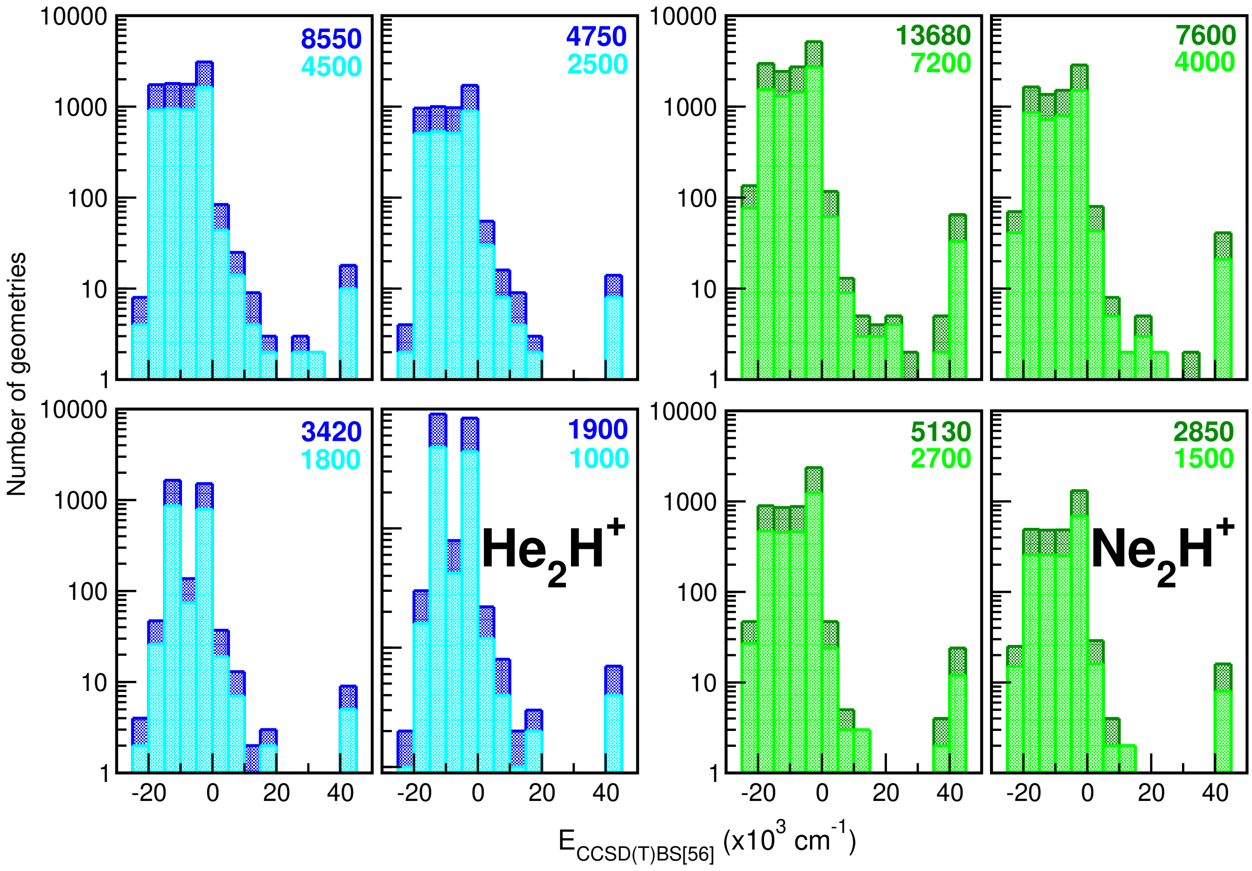

Figure S1: Histograms of different size training data set employed in building up the RHKHS ML-PES models for $[He_2H]^+$ (left blue panels) and $[Ne_2H]^+$ (riht green panels) complexes.

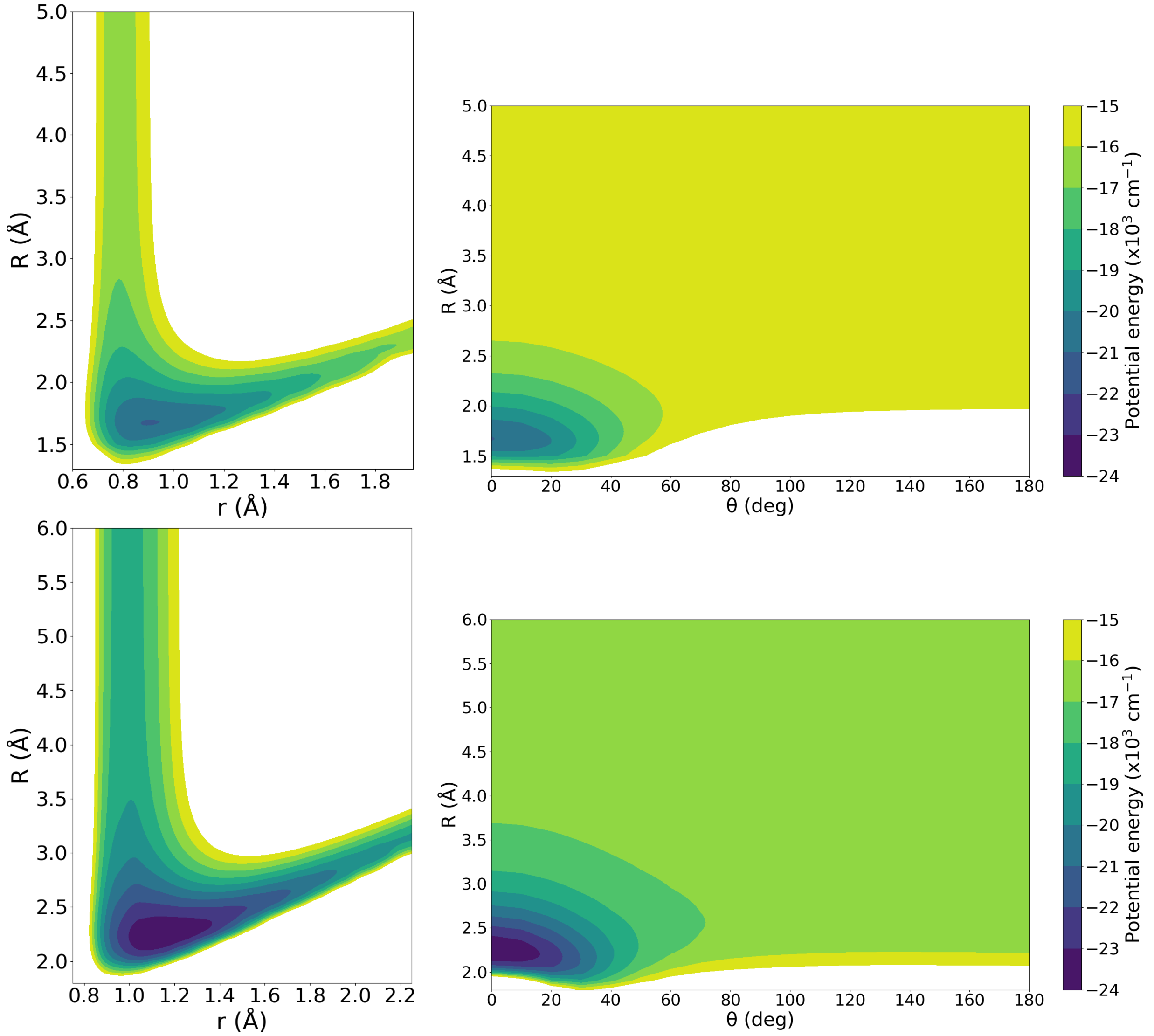

Figure S2: Contour plots of the He_2H^+ (upper panels) and Ne_2H^+ (lower panels) RKHS ML-PESs in the (r, R) (left panels) and (θ, R)-planes (right panels) with θ=0 °, and r=0.95 and 1.15 Å respectively. Potential energy values are given in cm^{-1}.
